# Supplementary material for: Cytoskeletal Protein 4.1G Is Essential for the Primary Ciliogenesis and Osteoblast Differentiation in Bone Formation
Source: Int J Mol Sci. 2022 Feb 14;23(4):2094. doi: 10.3390/ijms23042094 (PMC8878336; doi:10.3390/ijms23042094)
Supplement: Supplementary file 1 [file ijms-23-02094-s001.zip › ijms-1552237-supplementary.pdf]

Supplementary data

Cytoskeletal protein 4.1G is essential for the primary ciliogenesis and osteoblast differentiation in bone formation

Masaki Saito, Marina Hirano, Tomohiro Izumi, Yu Mori, Kentaro Ito, Yurika Saitoh, Nobuo Terada, Takeya Sato, Jun Sukegawa

Table S1. Primer sequences

Primer name	Sequence
4.1G Wt-Neo Both Left1	GTGGTCTTGAGTTGGCTGTG
4.1G Wt Right2	TTATCCCAAACCAGCCAGTC
4.1G Neo Right1	TCGCCTTCTTGACGAGTTCT
SX-F	GATGATTTGAGTGGAAATGTGAGGTA
SX-R	CTTATGTTTATAGGCATGCACCATGTA
4.1G-F	GGAAGTCCCCATTGTTCAAA
4.1G-R	AGAGCTGCGTCTCCTGTGAT
Gli1-F	GGTGCTGCCTATAGCCAGTGTCCTC
Gli1-R	GTGCCAATCCGGTGGAGTCAGACCC
Ptch 1-F	CTCTGGAGCAGATTTCCAAGG
Ptch 1-R	TGCCGCAGTTCTTTTGAATG
Osterix-F	AGCGACCACTTGAGCAAACAT
Osterix-R	GCGGCTGATTGGCTTCTTCT
Osteocalcin-F	AAGCAGGAGGGCAATAAGGT
Osteocalcin-R	ACTTGCAGGGCAGAGAGAGA
Runx2-F	ACAACCACAGAACCACAAG
Runx2-R	TCTCGGTGGCTGGTAGTGA
GAPDH-F	AGGTCGGTGTGAACGGATTTG
GAPDH-R	TGTAGACCATGTAGTTGAGGTCA

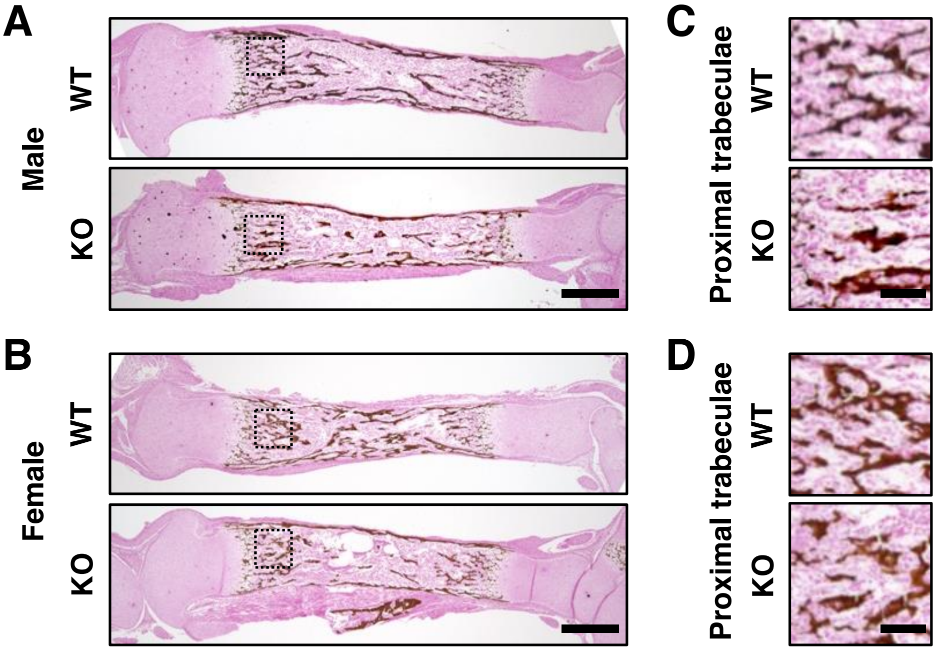

Figure S1. Protein 4.1G promotes bone formation at the trabecular bone in newborn tibia. The newborn tibia sections from the male (**A, C**) and female (**B, D**) mice were stained by modified von Kossa's reaction. (**A**, **B**) Images are the same as the images of figures **1A** and **1B**. (**C**, **D**) Enlarged views of the boxed areas in **A** and **B**, respectively. Scale bars, 500 μm (**A**, **B**) and 100 μm (**C**, **D**).

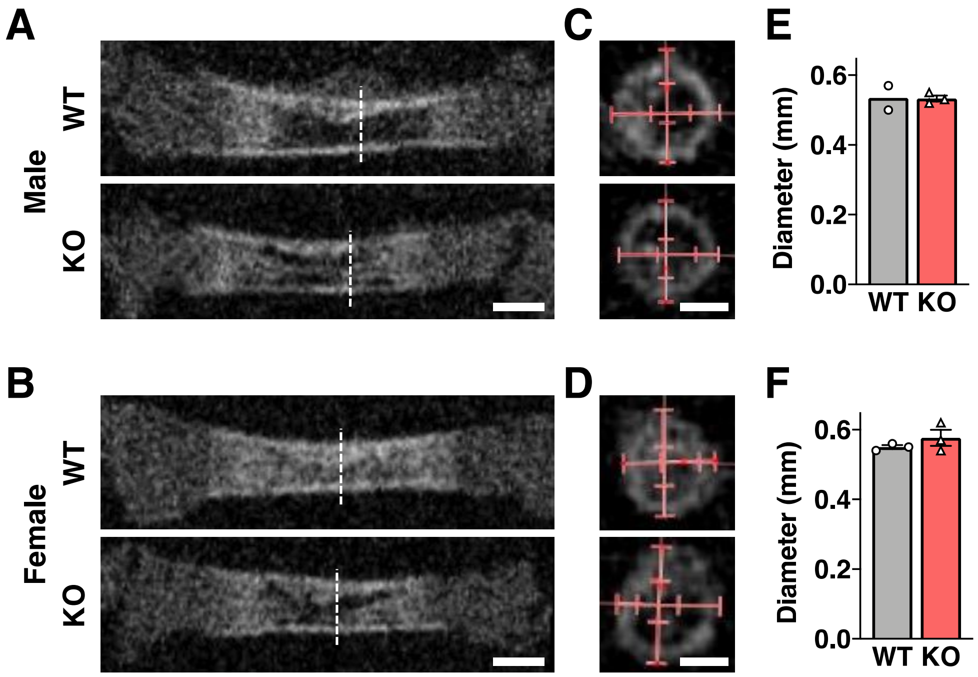

Figure S2. Protein 4.1G has no effect on the tibia diaphyseal diameter. (**A, B**) Micro-CT images of the male (**A**) and female (**B**) mice. (**C, D**) Cross-section images obtained at white dashed bars in (**A**) and (**B**) were shown, respectively. Two transverse diameters that cross at a right angle were measured (illustrated as the red lines), and the longer diameter was applied for the quantification. (**E**, **F**) Quantified diameter. Scale bars, 500 μm (**A**, **B**) and 300 μm (**C**, **D**).

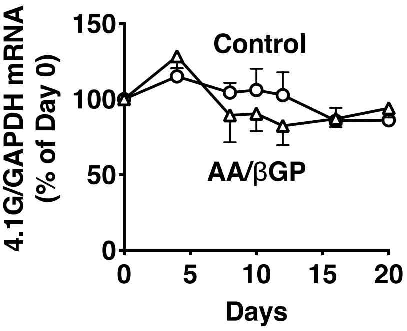

Figure S3. mRNA expression of 4.1G is not changed during the differentiation of MC3T3-E1 cells. The confluent MC3T3-E1 cells were treated with (triangle) or without (circle) 250 μM ascorbic acid and 50 μM β-glycerol phosphate (AA/βGP) for up to 20 days. mRNA expression of 4.1G was analyzed by RT-qPCR. mRNA content of 4.1G was normalized to glyceraldehyde-3-phosphate dehydrogenase (GAPDH). Data are presented as the mean ± standard error of the mean (S.E.M.) from three independent experiments.

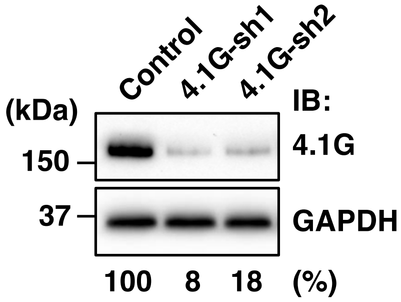

Figure S4. Knockdown efficiency of 4.1G-shRNA1 (4.1G-sh1) and 4.1G-shRNA2 (4.1G-sh2) in MC3T3-E1 cells. The whole cell lysates were immunoblotted (IB) with anti-4.1G or anti-GAPDH antibody. One of two independent representative immunoblots was shown. The relative amount of 4.1G was normalized to GAPDH shown below the blots (%).

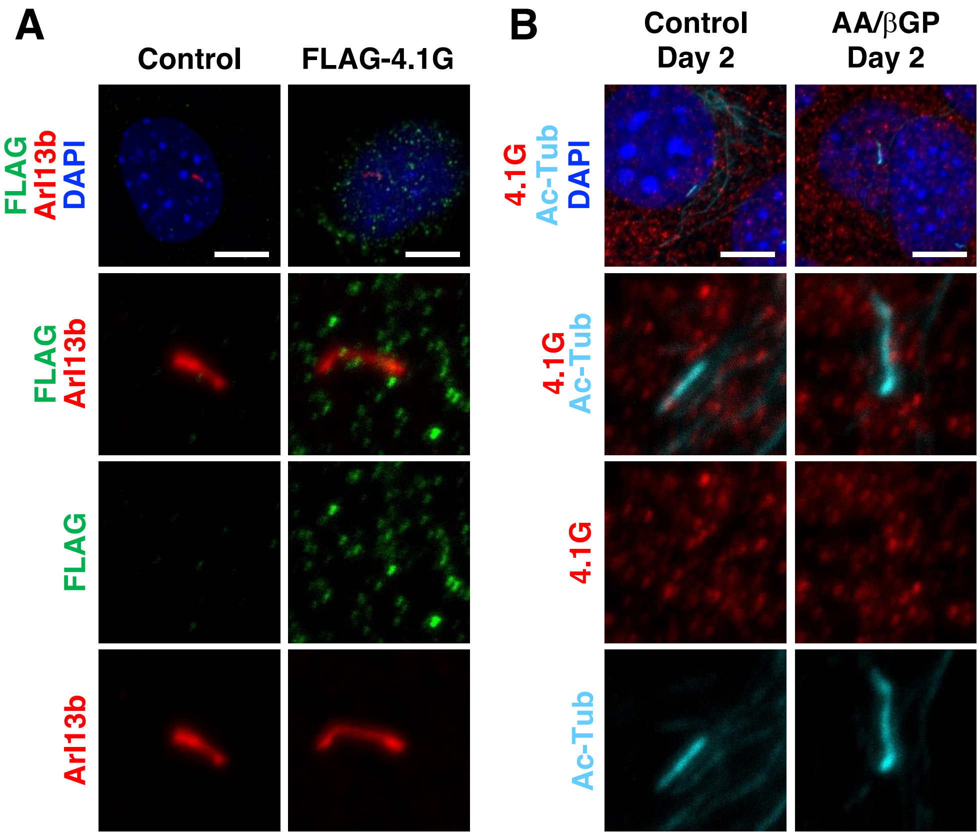

Figure S5. 4.1G is not accumulated at primary cilium or periciliary region. (**A**) Distribution of exogenous 4.1G in MC3T3-E1 cells. MC3T3-E1 cells expressing FLAG-tagged 4.1G were co-immunolabeled with the anti-FLAG (green) and anti-Arl13b (red) antibodies. (**B**) Distribution of endogenous 4.1G in MC3T3-E1 cells. MC3T3-E1 cells treated with AA/bGP for 2 days were co-immunolabeled with the anti-4.1G (red) and anti-Ac-Tub (cyan) antibodies. The cell nuclei were labeled by DAPI. Scale bars, 10 μm.

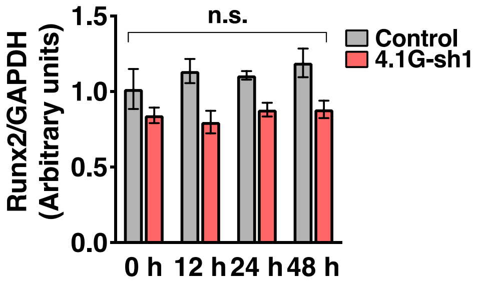

Figure S6. Runx2 mRNA level is not influenced by 4.1G expression. MC3T3-E1 cells transfected with control or 4.1G-sh1 were treated with AA/bGP for 2 days. The cells were further stimulated with 2 μM of purmorphamine at indicated time points. Relative mRNA expression of Runx2 was normalized to GAPDH. Data are presented as the mean ± S.E.M. from three independent experiments. n.s., not significant.
